# Facial Landmark-Driven Keypoint Feature Extraction for Robust Facial Expression Recognition

**DOI:** 10.3390/s25123762

**Published:** 2025-06-16

**Authors:** Jaehyun So, Youngjoon Han

**Affiliations:** 1 Department of Electronic Engineering, Soongsil University, Seoul 06978, Republic of Korea; ru2ror@soongsil.ac.kr; 2 School of AI Convergence, Soongsil University, Seoul 06978, Republic of Korea

**Keywords:** facial expression recognition, face alignment, deep neural network, feature attention

## Abstract

**Highlights:**

**What are the main findings?**

- We proposed Keypoint Features, a novel approach for feature representation based on facial landmarks, and NKF, a framework for robust FER, which was designed for clear component analysis.
- We demonstrated state-of-the-art FER performance on benchmark datasets and performed a rigorous analysis of component contributions via ablation studies, and demonstrated strong generalization.

**What are the implications of the main findings?**

- This work demonstrates the significant potential of keypoint-based FER, paving the way for its application in real-world scenarios and potential extension to other landmark-driven tasks.
- This work provides a strong empirical foundation and performs an analysis of the components that can guide the future research in developing highly accurate and computationally efficient FER systems for unconstrained environments.

**Abstract:**

Facial expression recognition (FER) is a core technology that enables computers to understand and react to human emotions. In particular, the use of face alignment algorithms as a preprocessing step in image-based FER is important for accurately normalizing face images in terms of scale, rotation, and translation to improve FER accuracy. Recently, FER studies have been actively leveraging feature maps computed by face alignment networks to enhance FER performance. However, previous studies were limited in their ability to effectively apply information from specific facial regions that are important for FER, as they either only used facial landmarks during the preprocessing step or relied solely on the feature maps from the face alignment networks. In this paper, we propose the use of Keypoint Features extracted from feature maps at the coordinates of facial landmarks. To effectively utilize Keypoint Features, we further propose a Keypoint Feature regularization method using landmark perturbation for robustness, and an attention mechanism that emphasizes all Keypoint Features using representative Keypoint Features derived from a nasal base landmark, which carries information for the whole face, to improve performance. We performed experiments on the AffectNet, RAF-DB, and FERPlus datasets using a simply designed network to validate the effectiveness of the proposed method. As a result, the proposed method achieved a performance of 68.17% on AffectNet-7, 64.87% on AffectNet-8, 93.16% on RAF-DB, and 91.44% on FERPlus. Furthermore, the network pretrained on AffectNet-8 had improved performances of 94.04% on RAF-DB and 91.66% on FERPlus. These results demonstrate that the proposed Keypoint Features can achieve comparable results to those of the existing methods, highlighting their potential for enhancing FER performance through the effective utilization of key facial region features.

## 1. Introduction

Facial information is essential for enabling computers to understand human emotions in the field of human–computer interaction. In particular, facial expressions play a crucial role in understanding human affective states and intentions. Consequently, facial expression recognition (FER) methods have been actively studied, and their performance has been significantly enhanced due to rapid advancements in deep learning technologies. FER has increasingly become recognized as a core technology in providing user-customized experiences across various industries.

The sensors used for FER can be broadly divided into contact and non-contact types. Contact sensors, such as those capturing brain wave or electromyography data, can be difficult to reliably attach onto users’ skin, which limits their practicality for everyday use. However, non-contact sensors, including cameras and microphones, can capture image and audio data from a distance. These sensors have significantly fewer constraints compared with contact sensors. In particular, most of the information humans perceive from external environments is visual data, and facial expressions are essentially a form of visual non-verbal communication. Therefore, an approach focused on analyzing facial expression using image data could be a direct and efficient method for FER.

However, FER in the wild remains challenging due to factors such as large facial poses and occlusions. Although FER methods typically process images of individual faces, such as ID photos, general images frequently contain various background elements. This makes the preprocessing steps that focus explicitly on the face crucial for performance. Generally, a FER pipeline involves detecting facial regions using a face detection algorithm and subsequently normalizing the detected face using a face alignment algorithm that performs scaling, rotation, and translation based on predicted facial landmarks. These preprocessing steps allow the FER network to learn consistent feature representations, leading to stable inference in various environments.

Recently, Zheng et al. [1] proposed a method utilizing feature maps computed from a face alignment network. Their study showed that face alignment networks could function not only as preprocessing tools but could also directly contribute to enhancing FER performance through leveraging these feature maps. Although these approaches are effective, the comprehensive analyses and structural designs remain insufficient for fully utilizing face alignment information. Prior studies using face alignment networks either as preprocessing tools or feature extractors have shown limitations in achieving high FER accuracy, indicating that the face alignment information was not fully exploited.

When humans recognize another person’s emotions, they tend to focus on specific facial regions, such as the eyes, nose, and mouth. Similarly, several FER studies [1,2,3,4] have shown that their networks focused on these specific facial regions through visualization methods and emphasized that these regions provided significant information for FER.

Motivated by these observations and the limitations of previous approaches, we propose a novel approach that introduces the use of Keypoint Features and a dedicated network architecture to leverage them for FER. These features are designed to fully exploit the rich information provided by the face alignment network, thereby enhancing FER performance. Our core contribution is the introduction of Keypoint Features, which are meticulously derived from semantically meaningful facial regions within the combined feature maps of a backbone network and a face alignment network. Their locations are precisely determined using the predicted facial landmark coordinates. This approach enables a more effective and comprehensive utilization of fine-grained information from specific facial regions, thus surpassing the limitations of methods that rely solely on landmark coordinates for image alignment or holistic feature map interpretation. To fully utilize and optimize the proposed Keypoint Features, we designed a Network for utilizing Keypoint Features (NKF). This network comprises two essential components: a novel Keypoint Feature regularization method based on landmark perturbation and Representative Keypoint Feature Attention (RKFA). Landmark perturbation-based regularization, applied during training, enhances the model’s robustness to potential inaccuracies in predicted facial landmarks and aims to mitigate the negative effects of imperfect landmark detection in the wild. RKFA adaptively emphasizes the most salient facial cues by leveraging a representative Keypoint Feature derived from the nasal base landmark, which encapsulates information for the entire face, thereby improving FER performance.

**Figure 1:**
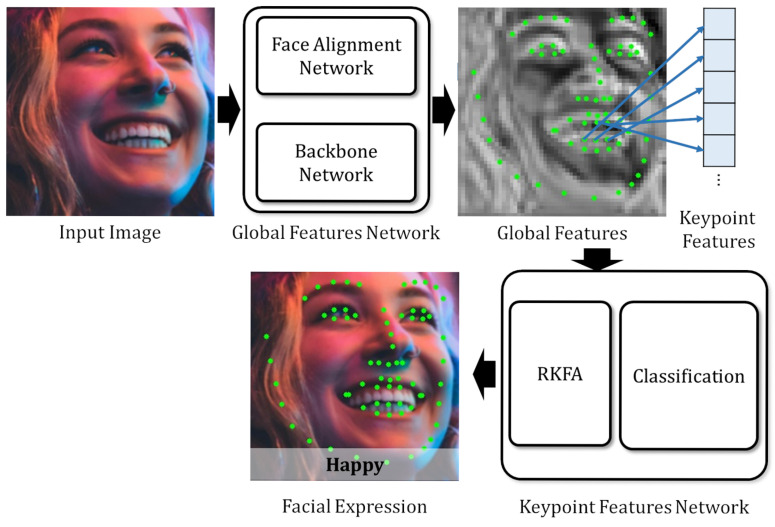
Flow of the proposed FER method. Global features are computed from the backbone and face alignment networks. Keypoint features are then extracted from these global features based on facial landmark coordinates from the face alignment network, followed by refinement using Representative Keypoint Feature Attention, and finally, classification.

Figure 1 illustrates the NKF’s flow. NKF primarily consists of a Global Feature Network and a Keypoint Feature Network. From an input face image, the Global Feature Network obtains feature maps from the backbone network. Simultaneously, it retrieves feature maps and predicts facial landmark coordinates from the face alignment network. These feature maps are then concatenated to produce global features. Subsequently, the Keypoint Feature Network extracts Keypoint Features from the global features based on the predicted facial landmark coordinates. The RKFA module then refines the Keypoint Features using a nasal base Keypoint Feature that contains information for the whole face. Finally, the network classifies the refined Keypoint Features for FER.

To validate the effectiveness of the proposed method, we evaluated it on widely adopted FER benchmark datasets, including AffectNet [5], RAF-DB [6], and FERPlus [7]. These datasets consist of facial images captured using standard camera sensors used in image-based FER studies.

The experimental results using standard protocols demonstrate that our method achieves a performance comparable to previous methods in these benchmarks. Specifically, we designed the NKF with a simple structure to clearly analyze the effectiveness of the proposed components and validated it through quantitative evaluations and ablation studies. In this study, we do not propose a novel sensor or a new dataset, but rather focus on developing a novel computational methodology for analyzing the image data produced by camera sensing technologies in the context of FER. Our primary contributions are as follows:

1. We propose a novel type of feature called Keypoint Features, which are extracted from feature maps at the coordinates of facial landmarks predicted by a face alignment network. This feature enables a more effective utilization of information from specific facial regions.
2. To enhance the quality of the extracted Keypoint Features, we introduce two methods: (i) Keypoint Feature regularization, which aims to reduce the negative effect of incorrectly predicted facial landmarks; and (ii) Representative Keypoint Feature Attention (RKFA), which uses a nasal base Keypoint Feature that includes whole-face information to improve FER performance.
3. We designed a deliberately simple network, excluding other advanced structures, to clearly analyze the effects of the proposed components. We empirically validated their contributions through experiments on multiple benchmark datasets and ablation studies.

## 2. Related Work

Facial expression recognition (FER) is a crucial area of affective computing. Broadly, approaches can be categorized into uni-modal and multi-modal methods. Uni-modal FER primarily relies on a single source of information, most commonly facial images, to recognize expressions. In contrast, multi-modal FER integrates diverse data streams for a more comprehensive understanding of emotions. For instance, recent advancements in multi-modal emotion recognition include systems that primarily rely on non-visual physiological signals, such as electroencephalography (EEG) and electrocardiography (ECG) [8]. However, acquiring such physiological signals often requires specialized close-proximity sensors, which can limit their applicability in daily scenarios. In contrast, other multi-modal approaches aim to improve the FER performance by leveraging combinations such as facial images and textual data to capture a richer emotional context [9,10]. While multi-modal methods are valuable for holistic emotion recognition, facial images inherently provide the most direct and rich source of information for facial expression recognition, as facial expressions are essentially a form of visual non-verbal communication. Thus, uni-modal, image-based FER methods remain critically important.

For facial expression recognition (FER), various methods have been proposed that leverage facial properties alongside standard deep network architectures, such as ResNet [11] and ViT [12], and attention modules like SE [13] and the self-attention mechanism of ViT. Because multiple facial expressions can appear on the same face, some studies [14,15] have aimed to emphasize expressive components while degrading facial identity. Other FER methods [16,17,18,19,20] have focused on loss functions designed to minimize intra-class distance and maximize inter-class separability in the expression feature space. Additional studies [21,22] addressed expression ambiguity by refining label distribution and feature representations. Because the proposed method focuses on utilizing face alignment for FER, we excluded the aforementioned methods that primarily address distribution alignment or representation learning to validate the contribution of face alignment more clearly.

Face alignment-based FER approaches can be broadly categorized based on their application in either the preprocessing or feature extraction stage. In our method, we incorporated face alignment into both stages. This section introduces previous face alignment-based FER methods and their limitations.

### 2.1. FER with Face Alignment as a Preprocessing Step

Most FER datasets focus on the expressions of individual faces. In general, a face image is cropped using a face detection method, and its scale, rotation, and translation are normalized using a face alignment method [23,24,25,26,27,28,29,30,31].

Most FER studies have employed face alignment in the preprocessing stage, which consequently limits the improvement in FER performance. Typically, the face alignment methods used for FER preprocessing have lower accuracy than more sophisticated modern methods. Commonly used methods include face detection-based approaches, such as MTCNN [32] and RetinaFace [33], or conventional algorithms, such as ERT [34], which utilize gradient boosting trees rather than deep neural networks.

### 2.2. FER with Face Alignment for Feature Extraction

Kollias et al. [35] fitted a 3D Morphable Model [36] using face alignment, subsequently generating synthetic-expression images based on the fitted model for use in FER. However, this method is limited because it only modifies the input images and does not directly enhance the feature maps of the FER network. Chen et al. [37] proposed a method to reduce expression ambiguity by constructing a landmark-based k-nearest neighbor graph that adjusts similar expressions to have similar label distributions. Liu et al. [38], following a strategy similar to that of Kollias et al., generated synthetic images while simultaneously training their network with landmark information via multi-task learning, enabling the network to suppress identity information. Ding et al. [39] enhanced the network’s feature extraction performance by generating attention maps derived from landmark locations. Furthermore, Zheng et al. [1], Mao et al. [2], and Chen et al. [40] employed MobileFaceNet [41], a face alignment network, to perform cross-attention between its generated feature maps and those from the backbone networks. However, these studies only utilized feature maps from the face alignment network, without leveraging landmark coordinates, thus limiting the potential benefits of face alignment.

In contrast, we propose a novel method that utilizes both feature maps and landmark coordinates from the face alignment network to enhance FER performance.

## 3. Proposed Method

**Figure 2:**
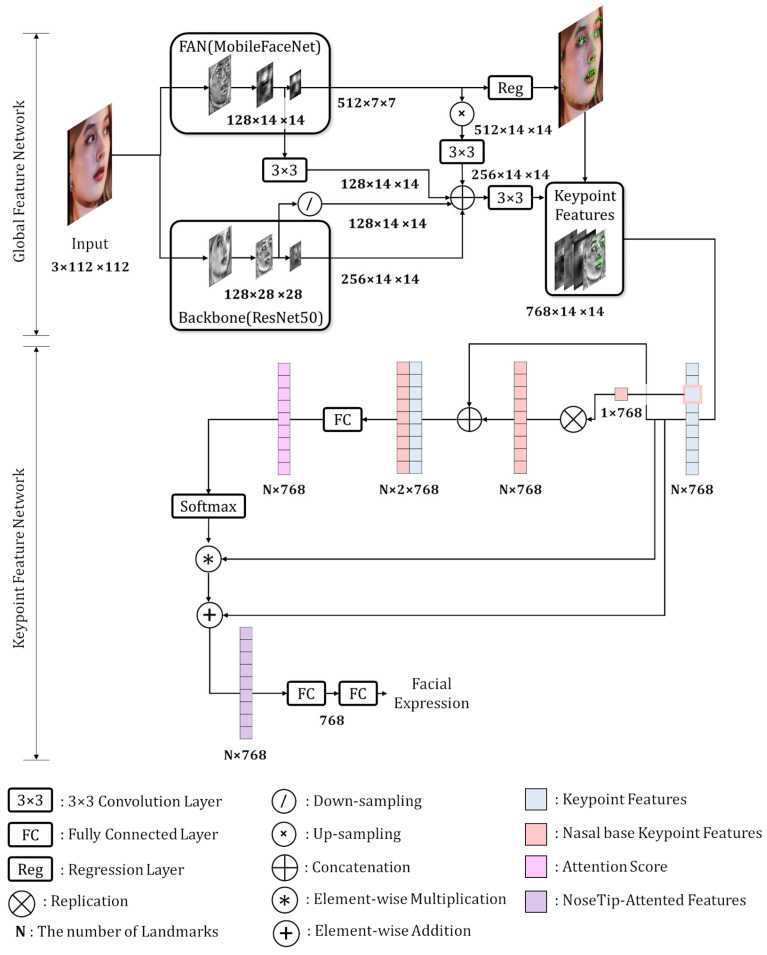
Overview of NKF structure for FER. The Global Feature Network detects facial landmarks and computes feature maps. The Keypoint Feature Network extracts the Keypoint Features from the Global Features at the facial landmark coordinates. Subsequently, they are improved by a Representative Keypoint Feature Attention module and finally classified for FER.

The structure of the proposed Network for utilizing Keypoint Features (NKF) is shown in Figure 2. This network consists of two main components: a Global Feature Network and a Keypoint Feature Network. The Global Feature Network includes a backbone network that generates its own feature maps and a face alignment network that generates feature maps and detects facial landmarks. These two types of feature maps are then fused to produce Global Features. The Keypoint Feature Network extracts the Keypoint Features from the Global Features based on the coordinates of the predicted facial landmarks and uses them to perform FER. 

Because this study focuses on strategies for utilizing Keypoint Features derived from face alignment, we briefly describe the relatively standard structure of the Global Feature Network and provide a more detailed explanation of the Keypoint Feature Network components in the following subsections.

### 3.1. Global Feature Network

The Global Feature Network generates feature maps, called global features, to perform FER. This network comprises a backbone network and a face alignment network to effectively extract facial information from input images. The backbone network computes feature maps dedicated to FER, whereas the face alignment network generates its own feature maps and predicts facial landmarks. These feature maps provide preliminary information for extracting Keypoint Features.

Because humans tend to recognize facial expressions based on facial shape rather than skin texture and color, it is crucial for a deep neural network used in FER to learn this characteristic to improve its performance. The feature maps from the face alignment network contain semantic information about facial landmarks, including the eyes, nose, and mouth, making them useful for understanding facial shape. Therefore, by fusing the feature maps from the face alignment network with those from the backbone network, rich information about the facial structure is incorporated into the combined feature maps.

Zheng et al. [1] and Mao et al. [2] improved FER performance through an attention mechanism, where feature maps from a face alignment network guided the backbone network’s attention toward facial landmarks. In contrast, our approach enhances FER performance by simply concatenating the feature maps from both networks. The fusion process is defined as Equation (1):

(1) $$F_{G}=F_{3\times 3,2}\left(D\left(F_{BBN}^{1}\right)⊕F_{BBN}^{2}⊕F_{3\times 3,0}\left(F_{FAN}^{1}\right)⊕F_{3\times 3,1}\left(U\left(F_{FAN}^{2}\right)\right)\right)$$

In Equation (1), $F_{3\times 3,i}$ represents the i-th 3 × 3 convolution layer, and $F^{j}$ denotes the feature maps of the j-th block layer. Here, BBN and FAN refer to the backbone and face alignment networks, respectively. D and U represent downsampling and upsampling operations, respectively, and ⊕ denotes feature map concatenation along the channel dimension. $F_{G}$ is the resulting global feature.

The dimensions of each feature map are as follows: $F_{BBN}^{1}$ has dimensions of 128 × 28 × 28 and is downsampled to 128 × 14 × 14. $F_{FAN}^{1}$ has dimensions of 128 × 14 × 14 and passed through $F_{3\times 3,0}$ without a change in size. $F_{FAN}^{2}$ has dimensions of 512 × 7 × 7, which is upsampled to 512 × 14 × 14 and then passed through $F_{3\times 3,1}$ to decrease the dimensions, resulting in 256 × 14 × 14. $F_{BBN}^{2}$ has the dimensions of 256 × 14 × 14. Their concatenation results in a 768 × 14 × 14 feature map, which is then passed through $F_{3\times 3,2}$ to produce a global feature, $F_{G}$, with the same dimension. Each convolution layer in Equation (1) does not include a batch-normalization layer or an activation layer. Both networks utilize residual block-based architectures, and the Global Feature Network exploits multi-level features to capture both low- and high-level semantic information, leading to better FER performance.

### 3.2. Keypoint Features

The Keypoint Feature Network extracts Keypoint Features from the previously generated global features, $F_{G}$, and utilizes them for FER. Facial landmark coordinates correspond to specific facial regions, such as the eyes, nose, and mouth, which are critical for FER. Siqueira et al. [4] utilized Grad-CAM [42] to demonstrate that the feature regions around these landmarks significantly influence FER. Recent ViT-based studies [1,2,3] have demonstrated similar findings. This suggests that features extracted from landmark coordinates are highly informative in FER. Keypoint Features are defined as follows:

(2) $$F_{K}=F_{G}\left(\left\lfloor x_{lmk}\right\rfloor \right),\;\;x_{lmk}=\left(x_{lmk},\;y_{lmk}\right)\;\;\;\;$$

where $x_{lmk}$ denotes the facial landmark coordinates predicted by the face alignment network. The Keypoint Features $F_{K}$ are sampled from the global features $F_{G}$ at these coordinates. Each of the 68 landmarks provides a 768-dimensional feature vector, resulting in $F_{K}$ with a size of 68 × 768. Because landmark coordinates are floating-point values, they are typically quantized to integer grid indices for direct indexing. However, this can introduce quantization errors that degrade feature quality. To address this, we employed a bilinear interpolation:

(3) $$F_{K}=f_{bilin}\left(F_{G},\;x_{lmk}\right)$$

where $f_{bilin}$ represents the bilinear interpolation function.

**Figure 3:**
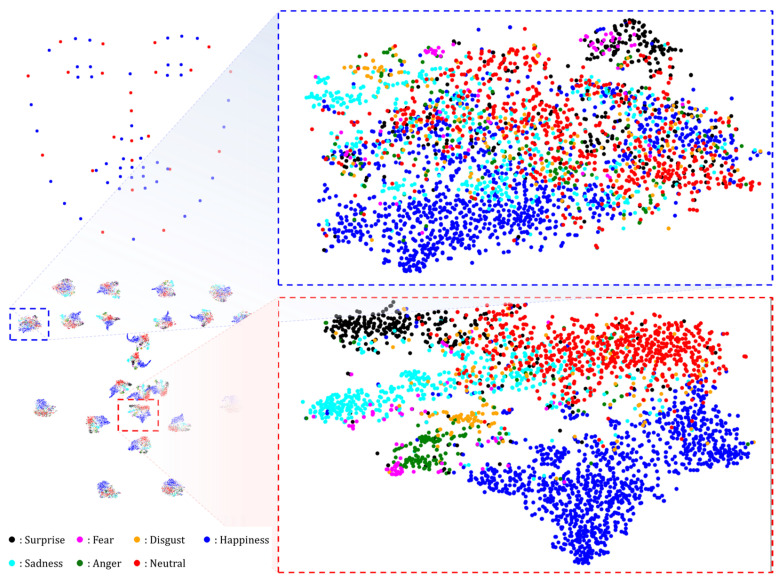
t-SNE-based [43] 2D projection of Keypoint Features extracted from test dataset samples after training the proposed network on the RAF-DB training set. The points at the top left represent the locations of the 68 mean landmarks of the 3D Morphable Model [36]. The projection results at the top right display only red dots, excluding blue dots for ease of visual analysis, and are placed at the corresponding locations. The blue box at the bottom shows an enlarged view of the distribution of the Keypoint Features corresponding to the outer face contour, and the red box shows an enlarged view of the distribution of the Keypoint Features corresponding to the upper lip.

Figure 3 shows the distributions of the Keypoint Features. Each landmark produced a distribution, totaling 68. The blue box in the figure highlights the Keypoint Features from the outer face contour, where ‘Happy’ and ‘Neutral’ expressions are relatively well-separated, while other expressions are harder to distinguish. In contrast, the red box illustrates the Keypoint Features around the upper lip, which exhibit clearer class separability. This suggests that mouth-region features are more discriminative for expression recognition than outer-contour features.

Although recognizing expressions based solely on facial landmarks is challenging, our Keypoint Features incorporate information about the entire facial region owing to the wide receptive fields obtained through the convolution layers of the Global Feature Network. Hence, even outer-region Keypoint Features contribute meaningfully, validating their overall effectiveness. A comparison of different Keypoint Feature types is presented in Section 4.3.2.

The final classification of the FER model employed two fully connected layers. After the first layer, batch normalization and a ReLU activation function were applied to generate 768 channel feature vectors, followed by a fully connected layer for classification.

### 3.3. Keypoint Feature Regularization

Since Keypoint Features are extracted based on facial landmark coordinates, they are inherently dependent on the accuracy of those coordinates. However, under in-the-wild conditions, landmark predictions can be imprecise owing to factors such as large pose variations or occlusions, which can degrade the FER performance. To mitigate this issue, we adopted a regularization method that introduced random perturbations to the landmark coordinates during training. Equation (4) illustrates how perturbed Keypoint Features are generated:

(4) $$\begin{array}{c} F_{K}=f_{bilin}\left(F_{G},\;x_{pred}+1_{train}\cdot U\left(-c,\;c\right)\right)\\ where\;1_{train}=\left\{\begin{array}{cc} 1, & \mathrm{if}\;\mathrm{training}\;\mathrm{phase}\;\\ 0, & else \end{array}\right. \end{array}$$

where U(−c,c) denotes a uniform distribution within the range [−c, c], and $1_{train}$ is an indicator function that equals 1 during training and 0 otherwise. During training, random offsets sampled from $U\left(-c,c\right)$ are added to the predicted landmark coordinates prior to feature extraction. By contrast, during inference, the original coordinates are used without perturbation. The hyperparameter c defines the maximum magnitude of the coordinate perturbation and can be tuned to optimize the robustness. This regularization method improves adaptability of the network to uncertainties in landmark detection and enhances its ability to generalize under various challenging conditions, such as diverse head poses and occlusions.

**Figure 4:**
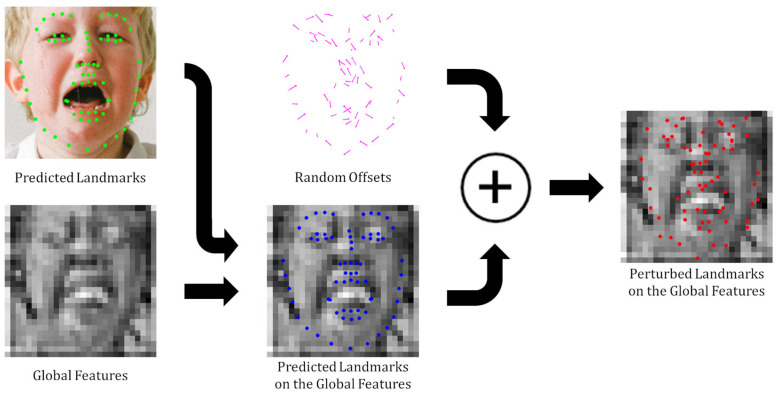
Generating inaccurately perturbed landmarks by adding random offsets during the training phase. The intentional incorporation of these inaccuracies in the landmark coordinates during training serves to extract Keypoint Features and enhance the network’s robustness and generalization performance. This process effectively makes the network less sensitive to potential errors in landmark detection.

Figure 4 illustrates the process of Keypoint Feature regularization. During the training phase, random offsets are added to the landmark coordinates, thereby introducing intentional noise into the Keypoint Features. This method generates various inaccurate Keypoint Features to provide a regularization effect and improve the network’s generalization performance on noisy data. Section 4.3.2 presents a performance comparison across different combinations of the proposed components, confirming that Keypoint Feature regularization contributes to the FER performance gains.

### 3.4. Representative Keypoint Feature Attention Using the Nasal Base Landmark

In Section 3.2, we analyzed the distribution of Keypoint Features and observed that some, particularly those located along the outer facial contour, exhibited a relatively low classification performance. Nevertheless, these features still encode unique and valuable information, and discarding them entirely may lead to suboptimal results. To address this, we propose Representative Keypoint Feature Attention (RKFA), a mechanism that enhances the classification performance of all Keypoint Features while preserving their contributions.

The proposed RKFA approach leverages the Keypoint Features at the nasal base landmark, which is located near the geometric center of the face. Features extracted from this location effectively captured the global facial structure and consistently demonstrated a strong classification performance. Therefore, by leveraging this globally representative feature, we aim to enhance all other Keypoint Features.

RKFA is implemented as a residual attention module inspired by conventional attention methods [13,44,45], with a key difference in that it duplicates the nasal base Keypoint Feature and broadcasts it across all Keypoint Features to compute attention weights. The RKFA mechanism is formulated as follows:

(5) $$F_{R}=\left(F_{K}+1\right)\times \mathrm{Softmax}\left(F_{fc}\left(\left(1⊗F_{K_{Nasalbase}}\right)⊕F_{K}\right)\right),\;1\in R^{N\times 1}\;$$

In Equation (5), $F_{K_{Nasalbase}}$ denotes the Keypoint Features at the nasal base landmark. The operator ⊗ replicates this feature N times, where N is the number of landmarks. The duplicated feature is concatenated with the full set of Keypoint Features, $F_{K}$, along the landmark dimension. This concatenated tensor is passed through a fully connected layer, $F_{fc}$, to compute the individual attention scores. The scores are normalized via a SoftMax function to produce attention weights, which are then applied channel-wise to refine each Keypoint Feature. The resulting features, $F_{R}$, are thus adaptively reweighted according to their importance, guided by the globally representative nasal base features.

**Figure 5:**
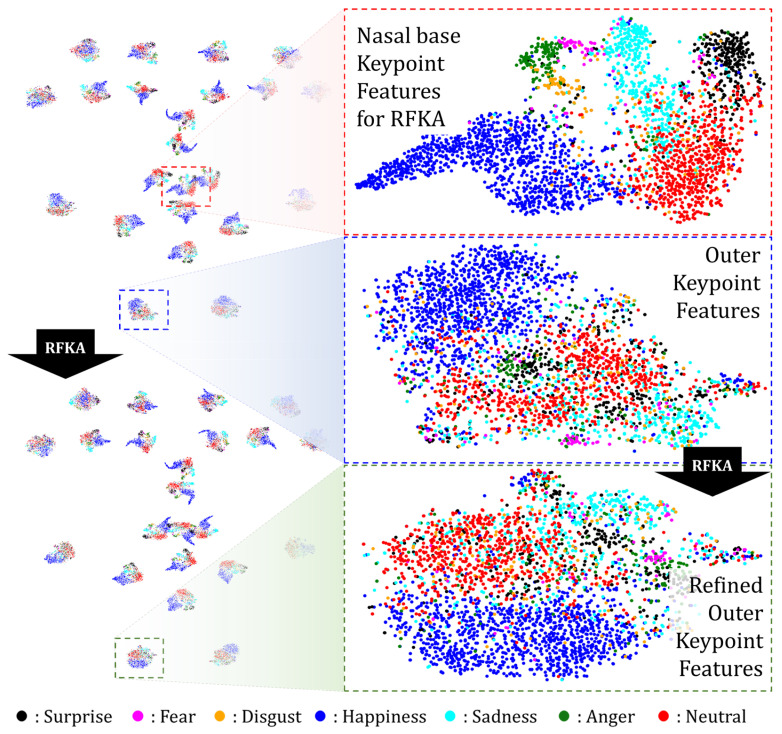
t-SNE [40] 2D projection illustrating Keypoint Feature distributions for samples from the RAF-DB test dataset, pre- and post-RKFA. The upper distribution shows the state before RKFA, and the lower distribution shows the state after RFKA is applied, all of which are depicted on the left side. The red, blue, and green boxes show the enlarged distribution of nasal base Keypoint Features, face outline Keypoint Features, and refined Keypoint Features after applying RKFA, respectively.

Figure 5 shows the distributions of the Keypoint Features before and after applying RKFA. Notably, the nasal base Keypoint Feature demonstrates relatively clear class separability even prior to applying the attention mechanism, whereas Keypoint Features located along the facial contour show more ambiguous class boundaries. After applying RKFA, the distribution of these outer Keypoint Features becomes more structured, and class separation improves significantly.

This improvement highlights the effectiveness of the RKFA mechanism in enhancing the discriminative power of all Keypoint Features, including those with initially weaker representations. The performance gains resulting from RKFA are further validated experimentally in Section 4.3.2, which demonstrates its positive contribution to overall FER accuracy.

## 4. Experiments

This section evaluates the proposed NKF introduced in Section 3. We assessed the accuracy of NKF and compared it with the state-of-the-art methods using widely adopted FER benchmark datasets, including AffectNet [5], RAF-DB [6], and FERPlus [7]. Furthermore, we validated the effectiveness of each proposed component through ablation studies.

### 4.1. Dataset

AffectNet is currently one of the most widely used large-scale datasets for facial expression recognition, with a training set of 287,651 samples and a test set of 4000 samples. The dataset provides both categorical labels (e.g., basic facial expressions) and dimensional labels (valence–arousal), along with 68 facial landmark annotations. Performance evaluations on categorical labels are typically performed under two settings: AffectNet-7, which includes seven categories (neutral, happy, sad, surprise, fear, angry, and disgust), and AffectNet-8, which adds the contempt category to the previous seven.

RAF-DB is another popular FER dataset containing 12,271 training samples and 3068 test samples. It includes annotations for 7 basic expressions (surprise, fear, disgust, happy, sad, anger, and neutral) and 12 compound expressions derived from combinations of these basic emotions. Each image was annotated by 40 human raters. The dataset provides 5 manually labeled and 37 automatically extracted facial landmarks, along with demographic metadata, such as race, age, and gender. In this paper, we evaluated the FER performance using only seven basic expression categories.

FERPlus is a revised version of the FER2013 [46] dataset, in which the original labels were re-annotated by 10 individuals per sample to correct prior inaccuracies. It consists of 28,709 training samples and 3589 samples for validation and testing. The dataset includes annotations for eight expressions (neutral, happy, surprise, sad, anger, disgust, fear, and contempt), as well as ‘unknown’ and ‘non-face’ labels. In our experiments, we evaluated the classification performance on the eight valid expressions, excluding the ‘unknown’ and ‘non-face’ classes. The ground truth labels were determined by majority voting across the 10 annotators.

### 4.2. Implementation Details

We trained a dedicated face alignment network to support the NKF pipeline. This network, based on MobileFaceNet [41], a lightweight architecture, was trained on each dataset individually. To guide its training, we utilized the PFA network [47], a high-accuracy face alignment model, to distill landmark knowledge. This approach ensures precise landmark detection and effective face alignment. The network was trained for 200 epochs across all datasets using stochastic gradient descent (SGD) with a learning rate of 0.01, momentum of 0.9, batch size of 16, and mean squared error (MSE) loss.

For the FER task, our training strategy followed a framework similar to POSTER++ [2]. The backbone network used for expression classification was ResNet50 (IR50) [48], pretrained on the Ms-Celeb-1M [49] dataset. The weights of the face alignment network were frozen during the FER training. The input images were resized to 112 × 112 via bilinear interpolation. We trained the FER model using the Sharpness-Aware Minimization (SAM) optimizer [50,51] built on top of Adam [52], with a batch size of 64, weight decay of 1.0 × 10^−4^, and an exponential learning rate decay of 0.98 per epoch. The cross-entropy loss function was used.

Data augmentation was performed in multiple stages as follows. First, we applied ±15% random scaling, ±30° rotation, ±15 pixels random translation, and 50% random horizontal flipping. Subsequently, we randomly selected and applied one of the following: Gaussian noise, Gaussian blur, grayscale conversion, contrast adjustment, color jittering, power law transform, histogram equalization, JPEG compression artifacts, lighting adjustment, or identity mapping. Finally, Random Erasing was applied to enhance model robustness through regularization.

All NKF models were trained using only the training split of each dataset. Because the AffectNet training set exhibited a class imbalance, whereas the test set was relatively balanced, we employed a class-balanced sampling strategy during training to mitigate this discrepancy. Specifically, samples from underrepresented classes were assigned higher sampling probabilities, whereas dominant classes were downweighted. However, when fine-tuning the AffectNet pretrained model on RAF-DB or FERPlus, we maintained the original class imbalance.

We implemented our models using Python 3.10 and the PyTorch 2.5 deep learning framework. All experiments were conducted on a system equipped with an AMD Ryzen 7 7700X CPU (AMD, Santa Clara, CA, USA) and an NVIDIA RTX3090 Ti GPU (NVIDIA, Santa Clara, CA, USA), operating under Ubuntu 22.04 LTS.

The training details for each dataset are summarized in Table 1. The landmark perturbation value, denoted as c in Equation (4) of Section 3.3, was multiplied by the size of the feature map to control the perturbation scale. A smaller value leads to a less significant impact on the landmark coordinates, whereas a larger value increases the regularization strength, improving robustness and generalization.

### 4.3. Evaluation

#### 4.3.1. Comparison with State-of-the-Art Methods

To validate the effectiveness of the proposed method, we compared the performance of our NKF framework with state-of-the-art FER methods on the AffectNet, RAF-DB, and FERPlus benchmark datasets. As shown in Table 2, the proposed NKF consistently achieves higher classification accuracy across all datasets and outperforms the existing state-of-the-art methods, thereby validating its superiority for FER tasks.

Table 2 compares the methods pretrained on AffectNet-8 with those that were not. The proposed NKF outperforms most existing state-of-the-art methods, except for its performance on the AffectNet-7 setting. Specifically, NKF achieved an accuracy that was 0.52% lower than that of Norface [38] on AffectNet-7 but surpassed DDAMFN [30] by 0.62% on AffectNet-8, Norface by 0.19% on RAF-DB, and S2D by 0.43% on FERPlus.

Norface utilizes additional generated images for training, which allows it to show an excellent performance on datasets such as AffectNet-7, where facial expressions are complex. Indeed, Norface demonstrated superior results on AffectNet-7, whereas NKF, trained without generated images, performed relatively worse on the same dataset. However, the proposed NKF, trained using only the images from the given dataset without generated images, achieved higher accuracy than Norface on the RAF-DB dataset, despite its comparatively worse performance on AffectNet-7.

Furthermore, when comparing models pretrained on AffectNet-8, NKF outperformed S2D by 1.57% on RAF-DB and 0.49% on FERPlus. These improvements represent a 2.44-fold increase on RAF-DB and a 1.38-fold increase on FERPlus in terms of the relative gains achieved through fine-tuning compared to S2D. These results highlight the impact of consistent landmark-based alignment across datasets, which effectively reduces domain discrepancies and maximizes the benefits of pretraining.

**Figure 6:**
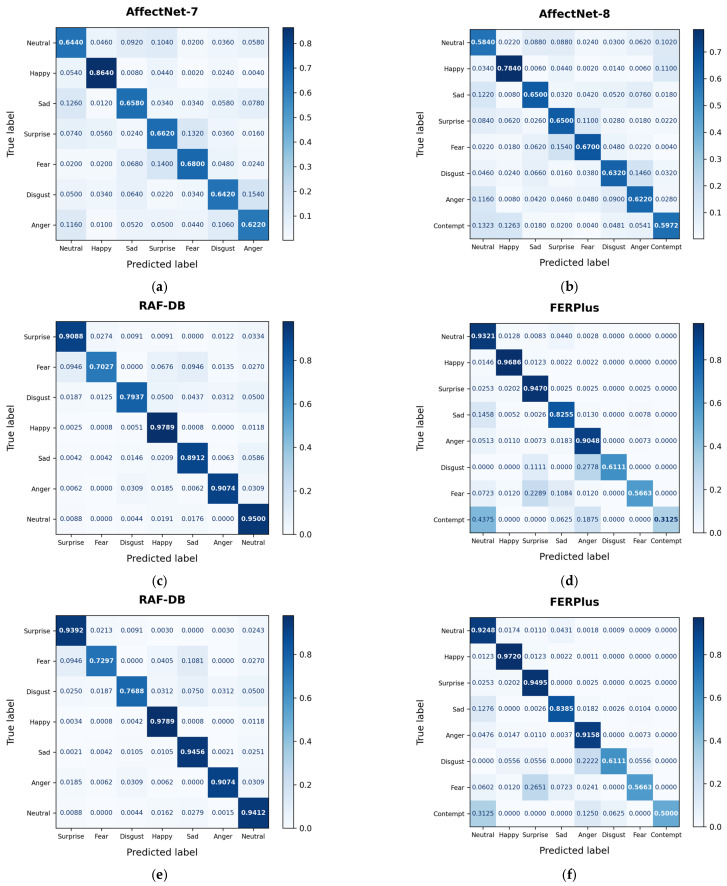
Confusion matrices of NKF evaluated on the FER datasets. (**a**) AffectNet-7 showing 68.17% accuracy of the NKF model, (**b**) AffectNet-8 showing 64.87% accuracy, (**c**) RAF-DB showing 93.12% accuracy, (**d**) FERPlus dataset showing 91.44% accuracy, (**e**) RAF-DB showing 94.04% accuracy pretrained on AffectNet-8, and (**f**) FERPlus showing 91.66% accuracy pretrained on AffectNet-8.

Figure 6 illustrates the classification performance of the proposed NKF on each dataset using confusion matrices, providing a detailed analysis of the prediction accuracy for individual expression classes. In all confusion matrices, except (d), each expression class shows a higher proportion of correctly classified samples than misclassified samples.

Although DDAMFN achieved a relatively high accuracy of 90.74% on the FERPlus dataset, it struggled significantly with the contempt class, frequently misclassifying it as neutral and anger. The proposed NKF also faces challenges in recognizing contempt; however, the NKF model pretrained on AffectNet-8 achieves the highest accuracy for this particular class, suggesting that the pretrained weights contribute positively to the recognition of nuanced expressions. Despite this improvement, the NKF’s accuracy for contempt remains lower than that for most other classes.

This lower accuracy is likely attributable to both the limited number of training samples for the contempt class and the subtle facial expression cues, which often resemble disgust or anger. These overlaps in visual features introduce ambiguity in neighboring classes, such as neutral, leading to frequent misclassifications. This trend, in which expressions with a less distinct visual separation from neutral are more prone to error, can also be observed for other ambiguous classes, such as disgust.

Table 3 compares the classification accuracy of NKF with that of state-of-the-art methods across individual expression classes for each dataset. In general, NKF demonstrates a higher overall accuracy than competing methods and consistently performs well, even in classes where other methods underperform. Although NKF occasionally shows slightly lower accuracy in certain high-performing classes compared to some specific approaches, it achieves the highest average accuracy overall.

These results highlight NKF’s strong generalization capabilities and the robustness of NKF in handling challenging expression classes, reinforcing its effectiveness as a reliable FER framework across varying datasets and emotional categories.

Additionally, we conducted a detailed attribute analysis of its performance on the RAF-DB dataset to evaluate its robustness and generalization capabilities. As presented in Table 4, the NKF generally shows a consistently good performance across gender, race, and age groups.

The NKF achieved over 90% accuracy for all groups, except for older adults aged 70+ years. This includes consistent accuracy across male, female, and ‘Unsure’ gender categories, as well as a good performance across all racial subgroups.

However, a decrease in performance was observed for older adults aged 70+ years, with accuracy falling into the 80% range. This decline is primarily attributed to age-related morphological changes, such as the development of wrinkles and skin folds, which can make the appearance of expressions ambiguous [58]. These physical alterations can make it more difficult for image-based FER systems to accurately interpret subtle cues of emotion in older faces.

This comprehensive attribute evaluation underscores the generalization capabilities of the NKF across varied populations and indicates the need for future improvement, particularly in enhancing performance for older-adult attributes.

**Figure 7:**
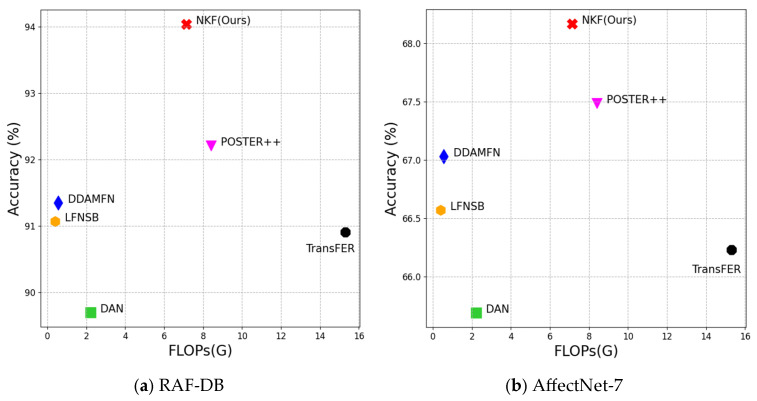
FLOPs versus accuracy: (**a**) depicts the accuracy on RAF-DB, and (**b**) depicts the accuracy on AffectNet-7. The red, pink, blue, yellow, black, and green symbols denote NKF, POSTER++ [2], DDAMFN [30], LFNSB [20], TransFER [56], and DAN [54], respectively.

Figure 7 compares the accuracies and FLOPs of various models on the RAF-DB and AffectNet-7 datasets. Although NKF incurs a relatively higher computational cost than lightweight architectures, it requires fewer FLOPs than POSTER++, despite using the same backbone [48] and face alignment networks [41]. Despite using fewer FLOPs, NKF achieves 94.04% accuracy with a 1.83% improvement on RAF-DB, 68.17% with a 0.68% improvement on AffectNet-7, and 64.87% with a 1.1% improvement on AffectNet-8.

For a more detailed comparative analysis, Table 5 presents the parameters, FLOPs, and accuracy across different models on the RAF-DB and AffectNet-7 datasets. In Table 5, ‘Global Net.’ refers to the combined computational cost of the backbone and face alignment networks, while ‘Local Net.’ denotes the proposed Keypoint Feature Network.

Although the NKF incurs a relatively higher computational cost than some lightweight architectures, Figure 7 demonstrates that the NKF demands fewer FLOPs than POSTER++, despite using the same backbone [48] and face alignment networks [41]. Regarding model complexity, the NKF generally exhibits a higher number of parameters than other models. This is primarily because we employed a straightforward fully connected layer to clearly demonstrate the effectiveness of the proposed Keypoint Features with a simple network structure. In particular, a substantial number of parameters are concentrated in the penultimate layer, which reduces the dimensionality from 68 × 768 to 768.

To further assess the practical efficiency of the NKF, we measured its inference speed. We report Frames Per Second (FPS) by distinguishing between the CPU and GPU times for full network inference. Our analysis suggests that both CPU-based inference on an AMD Ryzen 7 7700X CPU and GPU inference of the NKF on an NVIDIA RTX3090 Ti GPU are highly efficient, with both achieving real-time performance and indicating strong potential. On this hardware, our model achieved approximately 36.09 FPS for CPU inference and 244.62 FPS for GPU inference. Leveraging this efficiency, the NKF achieves 94.04% accuracy with a 1.83% improvement on RAF-DB, 68.17% with a 0.68% improvement on AffectNet-7, and 64.87% with a 1.1% improvement on AffectNet-8.

#### 4.3.2. Ablation Study

To comprehensively analyze the effectiveness of the NKF, we conducted a series of accuracy-based performance comparisons using the AffectNet-8 dataset. With its substantial number of samples and class-balanced evaluation, AffectNet-8 is a suitable benchmark for assessing the generalization ability of various models.

Our ablation analysis begins with an evaluation of the individual contributions of each proposed component through comparative experiments involving different network configurations. Each configuration incrementally integrates specific elements of the NKF framework. In addition, we assessed the influence of landmark types and the positioning of keypoints used in the Representative Keypoint Feature Attention (RKFA) mechanism.

Table 6 shows the performance of the NKF models designed by incorporating the various proposed components. In all cases, the input consisted of the original facial images provided in the dataset. The baseline model, which does not utilize any of these components, is constructed by applying global average pooling to feature maps of the backbone network to form a feature vector, followed by classification using a fully connected layer.

According to Table 6, applying accurate face alignment using facial landmarks yields a 1.03% performance improvement, raising the accuracy from 62.94% to 63.97%, thereby emphasizing the importance of precise facial alignment. The NKF model, which integrates all proposed components, achieves a total accuracy increase of 1.45% (from 62.94% to 64.39%), validating the cumulative effectiveness of the method. Moreover, we observed performance improvements across all configurations that included any of the proposed modules, whether applied individually or in combination.

Finally, when starting with aligned images as the input and then applying all proposed components, the model achieved an additional 0.90% gain, improving from 63.97% to 64.87%. This further confirms that the combined application of accurate alignment, Keypoint Feature extraction, regularization, and RKFA yields synergistic improvements in FER accuracy.

Keypoint Features are particularly effective owing to the wide receptive field of the feature maps computed by the underlying networks. Consequently, even when a limited number of landmark coordinates are used, each extracted feature can still capture information from a broad spatial area. This characteristic enables a robust FER performance despite the relatively sparse spatial sampling.

Facial expression cues are predominantly concentrated in specific regions of the face, namely the eyes, nose, and mouth. Accordingly, the critical information required for facial expression recognition can be sufficiently captured using Keypoint Features extracted only from these localized facial regions.

**Figure 8:**
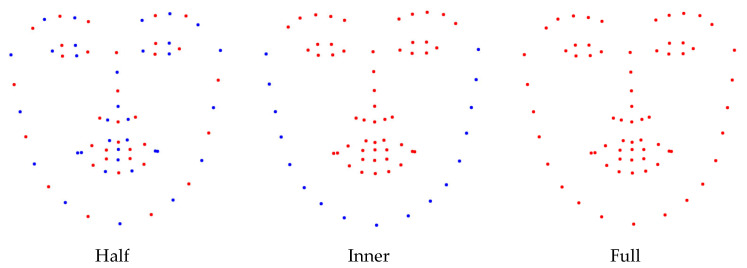
Three types of facial landmarks to extract Keypoint Features. Blue dots indicate unused landmarks; red dots indicate used landmarks. ‘Half’ denotes the selection of landmarks at odd-numbered indices. ‘Inner’ refers to the selection of landmarks within the face. ‘Full’ denotes the selection of all landmarks.

Figure 8 and Table 7 provide an overview of the different landmark subsets employed for Keypoint Feature extraction in this study. The results quantitatively demonstrate the efficacy of the proposed method by comparing FER performance across various landmark configurations, validating that key facial regions alone can serve as an effective basis for expression classification.

Table 7 presents the FER performance of the NKF models designed using different landmark configurations. The results demonstrate that FER remains feasible even when utilizing a reduced set of Keypoint Features, as evidenced by the ‘Half’ landmark type. However, this reduced configuration led to an overall drop in accuracy compared to the ‘Full’ landmark setting.

For the ‘Inner’ type, which comprises only landmarks located in the central facial regions, the results confirm that landmarks around the eyes, nose, and mouth are more informative than those on the facial periphery. Nonetheless, a slight performance degradation was observed relative to the ‘Full’ setting, indicating that the Keypoint Features along the outer face contour also carry useful expression-related information. This suggests that leveraging Keypoint Features from the entire face contributes to a higher FER accuracy and supports the holistic design of the proposed NKF framework.

The Representative Keypoint Feature Attention (RKFA) mechanism enhances FER by attending to the most expressive Keypoint Features. In this study, the nasal base Keypoint Feature was selected as the representative feature owing to its strong performance and central facial location. As this feature lies near the geometric center of aligned facial images—typically between the nose and mouth—it may be substituted with other centrally located features on the feature map without a significant loss of information.

**Figure 9:**
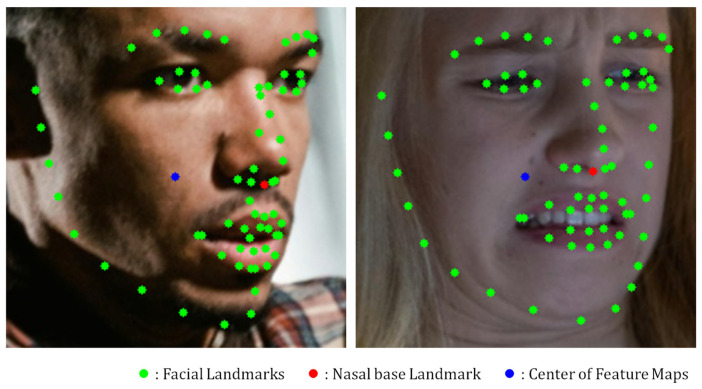
Examples of Keypoint Features. A green dot indicates a facial landmark, a red dot indicates a nasal base landmark, and a blue dot indicates the center location of a feature map.

Figure 9 provides a visual comparison between the general facial landmarks, nasal base landmark, and center point of the feature map. As illustrated, the nasal base landmark is situated very close to the center of the facial image, supporting its role as a representative anchor for attention computation in the RKFA. Table 8 presents a performance comparison of the NKF variants designed using different attention mechanisms. Using Keypoint Features based on the center point of the feature map improved the performance compared to the version without RKFA, indicating that even spatially central features can provide meaningful guidance.

Notably, the RKFA configuration that utilized the nasal base Keypoint Feature achieved the best performance among all attention types. This result can be attributed to the location of the nasal base landmark, which is positioned between the nose and mouth, capturing regions more directly associated with expressive facial movements than the geometric center point.

These findings suggest that, for enhancing the FER performance, the semantic relevance of Keypoint Features to expression representation is more critical than relying solely on their spatial proximity to the center of the face. In other words, attending to regions rich in expression-related cues yields more effective attention guidance than using position alone.

## 5. Discussion

We proposed a novel FER framework, NKF, which leverages Keypoint Features extracted from specific facial landmarks and integrates additional components to enhance recognition performance. Prior studies have demonstrated that facial regions, such as the eyes, nose, and mouth, carry highly discriminative cues for FER. In line with this, our method extracts Keypoint Features from these regions and uses them for effective expression classification. As shown in Figure 3, the proposed Keypoint Features form well-separated clusters across expression classes, validating their discriminative power.

Moreover, the landmark perturbation-based regularization improved the model’s robustness by addressing errors from inaccurately detected landmarks and enhancing generalization performance. The Representative Keypoint Feature Attention (RKFA) mechanism further contributed by refining lower-performing Keypoint Features using the nasal base Keypoint Feature, thereby maintaining expression-relevant information while improving the overall classification accuracy.

Although the nasal base Keypoint Feature proved effective as a representative cue for RKFA in our experiments, we are mindful that its universal representativeness might be challenged under certain conditions, such as extreme facial poses or severe occlusions. For large poses, such as profile faces, information from the nasal base landmark may be less effective; moreover, occlusion of this region could render the information entirely invalid. However, network weights trained on augmented large poses or occluded face images can potentially mitigate these limitations to some extent. While our current data augmentation strategy includes techniques such as Random Erasing for occlusion and 2D image rotation to introduce variations for large poses, it does not specifically address full profile faces. In such challenging scenarios, the perceived ‘holistic facial information’ of the nasal base could otherwise be distorted or obscured, potentially limiting the effectiveness of RKFA. Future work could explore more dynamic strategies for selecting representative landmarks, perhaps by employing a learned mechanism to adaptively choose the most reliable and informative reference point based on specific characteristics of the input image, including large pose and occlusion level, thereby enhancing the robustness of RKFA across a wider range of unconstrained facial images.

The proposed NKF, which integrates Keypoint Features, landmark perturbation, and RKFA, outperforms several state-of-the-art methods across multiple datasets. Specifically, NKF attained 64.87% accuracy on AffectNet-8 (+0.62% over DDAMFN [30]), 93.16% on RAF-DB (+0.19% over Norface [38]), and 91.44% on FERPlus (+0.43% over S2D [40]), outperforming several state-of-the-art methods. In particular, using aligned images based on precise facial landmarks significantly enhanced the performance and contributed to reducing the domain gap across datasets. Consequently, fine-tuning the AffectNet-8 pretrained model yielded 94.04% accuracy on RAF-DB (+1.57% over S2D [40]) and 91.66% on FERPlus (+0.49% over S2D [40]). Enhanced performance of the NKF is primarily attributed to several core factors: (1) high-quality Keypoint Features extracted from semantically meaningful facial regions, (2) robust regularization via landmark perturbation, and (3) targeted feature enhancement by RKFA. These results are consistent with prior works, such as POSTER [1], POSTER++ [2], EAC [3], and EfficientFace [4], which also emphasize the importance of region-specific features in FER.

In the AffectNet-7 evaluation, the NKF underperformed slightly compared to Norface [35] by 0.52%. This suggests that while the NKF is effective with the available dataset, it may face limitations when dealing with more complex or diverse data distributions without additional data augmentation. It is important to note that Norface incorporates synthetic images generated from an external dataset, creating a non-comparable training environment for the model.

To overcome these limitations, future work could explore the integration of the NKF with Vision Transformer (ViT)-based architectures or employ refined landmark definitions for more complex datasets. However, given that ViT lacks strong inductive biases, its effective application necessitates large-scale pretraining on datasets such as JFT [59] or ImageNet-21k [60]. Furthermore, the fixed patch structure of ViT may present compatibility challenges for landmark-based feature extraction. Moving forward, we aim to enhance performance by utilizing large-scale face-specific datasets and embedding self-attention mechanisms from ViT into the NKF. Such endeavors could extend the capabilities of keypoint-based FER and serve as a crucial step toward optimizing both recognition accuracy and computational efficiency in real-world scenarios.

**Figure 10:**
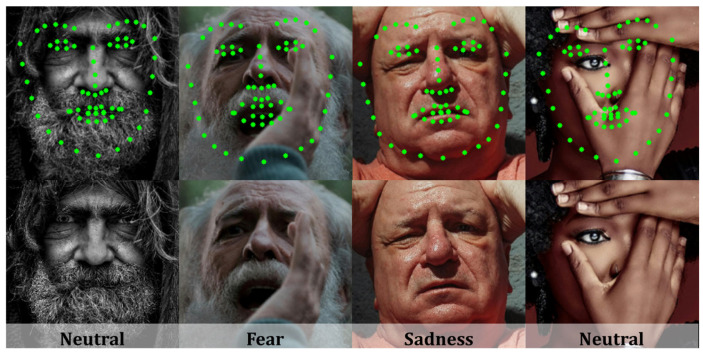
Examples of failed cases. The top row displays the input image with the overlaid predicted facial landmarks. The bottom row presents the same input image along with the predicted facial expression. The green dots denote the predictions of the face alignment network.

The NKF’s analysis of misclassified samples revealed specific challenging scenarios. As illustrated in Figure 10, we observed two primary contributing factors to recognition errors: inaccurately predicted facial landmarks and inherently ambiguous expressions.

The top row of Figure 10 presents examples of how factors, such as facial occlusion, can lead to mislocalized landmarks. Specifically, the first, second, and fourth columns illustrate faces with various forms of occlusion. Furthermore, in the third column, the eyebrow hair is nearly imperceptible, posing an additional challenge for accurate localization. These inaccurately localized Keypoint Features provide distorted facial information, which subsequently results in failures in downstream tasks.

Despite the facial landmarks being localized with relative accuracy in the first face shown in the bottom row, its FER result remains ambiguous. The first, second, and third examples in the bottom row feature ambiguous expressions, while the fourth example’s expression cannot be accurately recognized due to severe occlusion from a hand. Notably, even human perception struggles to guarantee accurate FER consistently in these challenging cases. Future work will consider an approach incorporating uncertainty-aware facial landmark prediction and FER.

## 6. Conclusions

In this paper, we introduce a novel facial expression recognition (FER) framework that leverages Keypoint Features derived from facial landmarks to enhance FER performance. Drawing inspiration from prior studies that emphasize the importance of specific facial regions, we extracted features from these landmark locations for expression classification. To further boost the effectiveness of the proposed Keypoint Features, we incorporated two techniques: landmark perturbation-based regularization, which improves robustness against detection noise, and Representative Keypoint Feature Attention (RKFA), which amplifies expressive regions using features from the nasal base landmark. As a result, NKF achieved impressive accuracies, attaining 64.87% on AffectNet-8, 93.16% on RAF-DB, and 91.44% on FERPlus. These results consistently outperform previous state-of-the-art methods. Furthermore, fine-tuning the NKF model pretrained on AffectNet-8 for RAF-DB and FERPlus led to additional performance improvements by reducing the domain gap between the datasets. Notably, the NKF demonstrated an excellent generalization ability, maintaining high accuracy even for difficult-to-recognize expressions, such as contempt. Despite these achievements, our approach has some limitations, including its reliance on the accuracy of face alignment and a relatively simple network architecture. Future work will address these issues by integrating global features via a Vision Transformer (ViT)-based structure and implementing a Keypoint weighting strategy that can estimate landmark uncertainty and increase resilience to occlusions and illumination variations. Through comprehensive empirical validation, we demonstrated the potential of Keypoint-based FER, laying a solid foundation for its extension to other landmark-driven tasks, such as human pose estimation. From an academic perspective, this work represents a meaningful step toward building FER systems that deliver a robust and high-accuracy performance in unconstrained real-world environments.

## Figures and Tables

**Table 1:** Settings of the difference between the datasets.

Parameters	AffectNet	RAF-DB	FERPlus
Initial Learning Rate	1.0 × 10^−6^	5.0 × 10^−6^	5.0 × 10^−6^
Epoch	50	200	200
Landmark Perturbation	0.05	0.10	0.5
Random Erasing Scale	(0.05, 0.05)	(0.02, 0.1)	(0.05, 0.05)

**Table 2:** Comparison with state-of-the-art methods. The best scores are in bold.

Method	Accuracy (%)			
	AffectNet-7	AffectNet-8	RAF-DB	FERPlus
KTN [53]	63.97	-	88.07	90.49
DAN [54]	65.69	62.09	89.70	-
EAC [3]	65.32	-	90.35	89.64
ARM [55]	65.20	61.33	90.42	-
TransFER [56]	66.23	-	90.91	90.83
LFNSB [20]	66.57	63.12	91.07	-
S2D [40]	66.42	63.76	92.21	91.01
EmoNeXt [57]	67.46	64.13	-	-
POSTER++ [2]	67.49	63.77	92.21	-
DDAMFN [30]	67.03	64.25	91.35	90.74
Norface [38]	**68.69**	-	92.97	-
NKF(Ours)	68.17	**64.87**	**93.16**	**91.44**
S2D † [40]	-	-	92.57 (+0.36)	91.17 (+0.16)
NKF † (Ours)	-	-	**94.04 (+0.88)**	**91.66 (+0.22)**

† means pretrained on AffectNet-8.

**Table 3:** Comparison with state-of-the-art methods for each facial expression. The best scores for each expression and dataset are in bold.

Data	Method	Accuracy (%)								
		Neu	Hap	Sad	Sur	Fea	Dis	Ang	Con	Avg
Aff7	KTN [53]	66.60	86.80	60.80	55.20	64.00	60.00	54.40	-	63.97
	ARM [55]	65.00	87.00	64.00	61.00	62.00	53.00	64.00	-	65.20
	LFNSB [20]	**68.79**	87.50	**69.44**	**66.67**	60.71	50.00	57.89	-	65.86
	POSTER++ [2]	65.40	**89.40**	68.00	66.00	64.20	55.40	**65.00**	-	67.45
	DDAMFN [30]	66.20	87.20	66.40	63.80	66.00	56.60	63.00	-	67.03
	NKF (Ours)	64.40	86.40	65.80	66.20	**68.00**	**64.20**	62.20	-	**68.17**
Aff8	ARM [55]	**63.00**	**86.00**	64.00	61.00	62.00	52.00	63.00	40.00	61.33
	LFNSB [20]	52.38	82.35	**68.75**	**80.00**	62.50	44.44	46.15	57.14	61.71
	POSTER++ [2]	60.60	76.40	66.80	65.60	63.00	58.00	60.20	59.52	63.76
	DDAMFN [30]	57.80	81.80	63.00	65.60	64.20	60.00	62.00	59.60	64.25
	NKF (Ours)	58.40	78.40	65.00	65.00	**67.00**	**63.20**	**62.20**	**59.72**	**64.87**
RAF	KTN [53]	88.53	94.60	87.24	83.28	68.92	65.62	81.48	-	81.38
	ARM [55]	**97.90**	95.40	83.90	90.30	70.30	64.40	77.20	-	82.77
	LFNSB [20]	92.79	96.71	87.24	87.84	67.57	74.38	87.65	-	84.88
	POSTER++ [2]	92.06	97.22	92.89	90.58	68.92	71.88	88.27	-	85.97
	DDAMFN [30]	92.49	96.60	89.55	91.03	65.75	72.33	84.57	-	84.62
	NKF(Ours)	95.00	**97.89**	89.12	90.88	70.27	**79.37**	**90.74**	-	87.61
	NKF † (Ours)	94.12	**97.89**	**94.56**	**93.92**	**72.97**	76.88	**90.74**	-	**88.73**
FERPlus	KTN [48]	92.07	95.85	81.38	92.96	**57.69**	53.33	90.41	30.77	74.31
	DDAMFN [30]	92.77	96.40	79.42	92.86	55.29	**62.50**	**93.23**	26.67	74.89
	NKF (Ours)	**93.21**	96.86	82.55	94.70	56.63	61.11	90.48	31.25	75.85
	NKF † (Ours)	92.48	**97.20**	**83.85**	**94.95**	56.63	61.11	91.58	**50.00**	**78.48**

† means pretrained on AffectNet-8.

**Table 4:** Performance of NKF on RAF-DB across gender, race, and age groups.

Attribute	Method	Accuracy (%)				
Gender	-	Male	Female	Unsure	-	-
	NKF (Ours)	91.83	93.77	95.98	-	-
	NKF † (Ours)	92.87	94.51	96.98	-	-
Race	-	Caucasian	African American	Asian	-	-
	NKF (Ours)	92.64	95.73	94.20	-	-
	NKF † (Ours)	93.32	97.01	95.86	-	-
Age	-	0–3	4–19	20–39	40–69	70+
	NKF (Ours)	96.96	94.24	92.36	93.23	86.52
	NKF † (Ours)	98.18	95.68	92.84	94.22	89.89

† means pretrained on AffectNet-8.

**Table 5:** Comparison of model complexity. Global Net. indicates the global networks and KF Net. indicates the Keypoint Feature Networks in the NKF. The best scores are highlighted in bold.

Method			#Param(M)	#FLOPs(G)	Accuracy (%)		
					RAF	Aff7	Aff8
TransFER [56]			65.20	15.30	90.91	66.23	-
DAN [54]			19.72	2.23	89.70	65.69	62.09
POSTER++ [2]			43.70	8.40	92.21	67.49	63.77
DDAMFN [30]			4.11	0.55	91.35	67.03	64.25
LFNSB [20]			2.68	0.38	91.07	66.57	63.12
NKF † (Ours)	Global Net.		25.24	7.01	-	-	-
	KF Net.	Penultimate Layer	40.11	0.04	-	-	-
		Others	1.19	0.08	-	-	-
	All		66.54	7.13	**94.04** †	**68.17**	**64.87**

† means pretrained on AffectNet-8.

**Table 6:** Evaluation of different components. The best scores are highlighted in bold.

Component		Choice						
Input	Original	✓	✓	-	-	-	-	-
	Accurately Aligned Image	-	-	✓	✓	✓	✓	✓
Network	Keypoint Features	-	✓	-	✓	✓	✓	✓
	KF Regularization	-	✓	-	-	✓	-	✓
	RKFA	-	✓	-	-	-	✓	✓
Accuracy (%)		62.94	64.39	63.97	64.44	64.59	64.57	**64.87**

**Table 7:** Evaluation of landmark types. The best scores are in bold.

Landmark Type	Accuracy (%)
Half, 34 landmarks	64.51
Inner, 51 landmarks	64.52
Full, 68 landmarks	**64.87**

**Table 8:** Effectiveness of the RKFA. The best scores are in bold.

Attention Type	Accuracy (%)
Without RKFA	64.59
Center point of the Feature Map	64.69
Nasal base	**64.87**

## Data Availability

Data were obtained from third parties and are available from the authors with the permission of the respective data providers. Specifically, AffectNet is available upon request and approval from the original authors at http://mohammadmahoor.com/pages/databases/affectnet/, accessed on 1 January 2025, and RAF-DB can be accessed with permission from http://www.whdeng.cn/RAF/model1.html, accessed on 25 October 2021. FERPlus is publicly available and can be accessed at https://github.com/microsoft/FERPlus accessed on 25 April 2025.
